# Characterization of the complete chloroplast genome sequence of *Hemsleya zhejiangensis* (cucurbitaceae), a rare and endangered wild plant species in Zhejiang province, China

**DOI:** 10.1080/23802359.2019.1692726

**Published:** 2019-11-18

**Authors:** Yanrong Li, Jinghui Li, Lingli Fang, Ming Jiang

**Affiliations:** Zhejiang Provincial Key Laboratory of Plant Evolutionary and Conservation, College of Life Science Taizhou University, Jiaojiang, China

**Keywords:** *Hemsleya zhejiangensis*, chloroplast genome, phylogenetic analysis

## Abstract

*Hemsleya zhejiangensis* is a rare and endangered plant species which is listed as a key protected wild plant in Zhejiang province, China. In our present study, we assembled the complete chloroplast (CP) genome of *H. zhejiangensis* using high-throughput sequencing data. The whole genome sequence of *H. zhejiangensis* is 157,289 bp in size, with a GC content of 37.1%. Sequencing analyses reveal that the CP genome encodes 133 genes, including 84 protein-coding genes, 8 rRNA genes, 37 tRNA genes, and four pseudogenes. Phylogenetic analysis results indicate that *H. zhejiangensis* is clustered with *H. lijiangensis*, with a support value of 100%, and they are sister to the three *Gynostemma* species.

The genus *Hemsleya* belongs to the family Cucurbitaceae and is comprised of about 27 plant species which are distributed in subtropical or tropical Asia (Wu et al. 2011). *Hemsleya zhejiangensis* is a plant with enlarged tubers, slender stems, and 5–9 foliolates. *Hemsleya zhejiangensis* is a wild plant species of extremely small population with fewer than 5000 individuals, and it is now listed as a key protected species in Zhejiang province, China. *Hemsleya zhejiangensis* is mainly distributed in counties of Taishun, Longyou, Jingning, and Yunhe, and it is used as a medicinal herb in fork medicine for treating enteritis, bacillary dysentery, coronary heart disease, and tracheitis (Zheng 1985; Yang et al. 2014). In our present study, we assembled the complete chloroplast (CP) genome of *H. zhejiangensis*, and phylogenetic analysis was performed to understand its relationship with other species.

Leaf samples were collected from Guanshan (27°53′42′′N, 119°31′51′′E), Yunhe county, Zhejiang province, China. A voucher specimen designated CHS2019022 is stored at the Molecular Biology Laboratory in Taizhou University. Genomic DNA isolation was performed following the CTAB-based method by Doyle and Doyle (1987), and a DNA library for high-throughput sequencing was then constructed following manufacturer's instructions. Totally, 6.8 Gb raw reads were generated using an Illumina Hiseq X Ten platform, and the clean reads were used for *de novo* assembly by a Perl program namely NOVOPlasty (Dierckxsens et al. 2017). Dual Organellar GenoMe Annotator (DOGMA) (Wyman et al. 2004) was applied to annotate the CP genome. tRNAscan-SE and ARAGORN were used for prediction of tRNAs (Lowe and Eddy 1997; Laslett and Canback 2004).

The results indicated that the whole CP genome of *H. zhejiangensis* (GenBank accession: MN414239) was 157,289 bp in length. The quadripartite structure was comprised of a large single copy, a small single copy, and two inverted repeats, and their sizes were 86,536 bp, 26,233 bp, and 44,520 bp, respectively. The CP genome encoded 133 genes, including 84 protein-coding genes, 37 tRNA genes, eight rRNA genes, and four pseudogenes (*psbC*, *psbL*, *infA*, and *ycf1*). Three protein-coding genes, *clpP*, *rps12*, and *ycf3*, had two introns, while other 16 genes (*atpF*, *ndhA*, *ndhB*, *petB*, *petD*, *rpl2*, *rpl16*, *rpoC1*, *rps12*, *rps16*, *trnA-UGC*, *trnG-UCC*, *trnI-GAU*, *trnk-UUU*, *trnL-UAA*, and *trnV-UAC*) contained only one intron.

To reveal the phylogenetic status with other related plant species, whole genome sequences of *Platycodon grandiflorus* (Campanulaceae) and 20 Cucurbitaceae plants were obtained from National Center for Biotechnology Information. The 20 Cucurbitaceae plants included species from genera of *Hemsleya*, *Gynostemma*, *Momordica*, *Siraitia*, *Coccinia*, *Lagenaria*, *Citrullus, Hodgsonia*, *Trichosanthes*, *Cucurbita*, *Cucumis*. A phylogenetic tree was generated by the maximum likelihood method (ML) using PhyML 3.1 (Guindon et al. 2010). The best DNA evolution model was GTR + G + I, which was computed by jModelTest 2 (Darriba et al. 2012). The phylogenomic analysis results revealed that the 22 plants were divided into 12 groups. *Hemsleya zhejiangensis* was closely related to *H. lijiangensis*, with a bootstrap support of 100%, and they were grouped with three *Gynostemma* species, with 100% bootstrap support (Figure 1).

**Figure 1.**
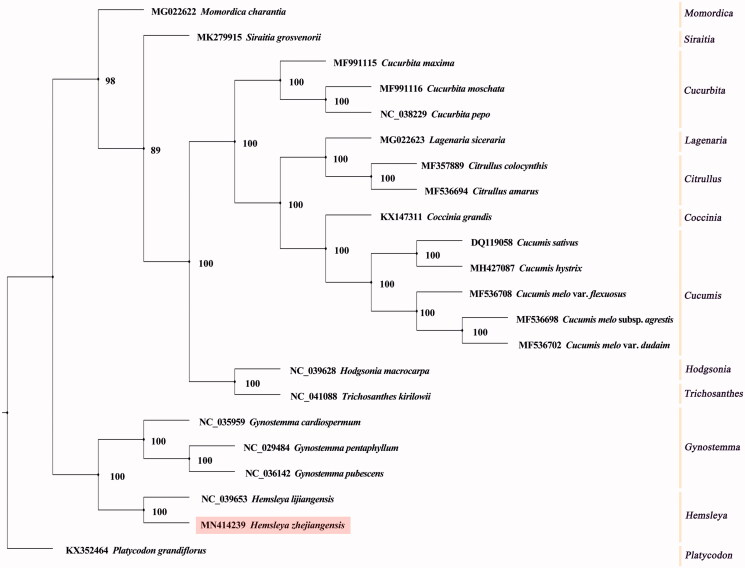
A maximum likelihood tree based on the complete chloroplast genome sequences of *Hemsleya zhejiangensis* and other Cucurbitaceae species, with *Platycodon grandiflorus* (Campanulaceae) as the outgroup. The numbers next to nodes are bootstrap support values.
